# Deterred but not preferred: Predation by native whelk *Reishia clavigera* on invasive bivalves

**DOI:** 10.1371/journal.pone.0196578

**Published:** 2018-05-16

**Authors:** Juan C. Astudillo, Timothy C. Bonebrake, Kenneth M. Y. Leung

**Affiliations:** 1 The Swire Institute of Marine Science, Faculty of Science, The University of Hong Kong, Shek O, Hong Kong, China; 2 School of Biological Sciences, The University of Hong Kong, Hong Kong, China; 3 State Key Laboratory in Marine Pollution, City University of Hong Kong, Kowloon, Hong Kong, China; University of California, UNITED STATES

## Abstract

This study tested the potential bio-control role of the common native predatory whelk *Reishia clavigera* on the invasive bivalves *Xenostrobus securis* and *Mytilopsis sallei* and the native *Brachidontes variabilis* in Hong Kong. Predation experiments were conducted in the laboratory under salinity levels of 22‰ and 32‰, as well as under field conditions. The results indicate that the invasive bivalves are more vulnerable to predation than the native bivalve in environments with high salinity, whereas environments with moderately low salinity (22‰) may reduce predation. Because *R*. *clavigera* did not show clear prey preference, the low survival of the invasive species might be due to a lack of effective anti-predatory defenses under experimental conditions. These findings could explain the high abundance of the invasive bivalves in disturbed environments in Hong Kong where predation appears to be lower.

## Introduction

Introduced species can establish and become invasive in novel environments if they are able to escape from natural enemies (predators, competitors, and parasites) that would otherwise limit their distribution and abundance [1–3]. Invasive species may find a spatial niche within native communities under unfavorable environmental conditions [4–6]. Similarly, estuaries and disturbed bays are known to be more susceptible to invasions than open and less disturbed coasts [7, 8]. Therefore, understanding the interactions between native predators and the environmental conditions on the survival of invasive species could provide insights into the control of their abundance and spread [9, 10].

Gastropods (grazer and predatory) are recognized for their strong impacts structuring benthic communities [11]. Few species have been tested as potential biological control agents on fouling communities that could eventually reduce the abundance and spread of invasive species [12]. Predatory gastropods from the subfamily Rapaninae present high diversity in the Indo-West Pacific [13, 14]. In the Northwestern Pacific, *Reishia clavigera* (Küster, 1860; formerly named as *Thais clavigera*) is one of the most common and abundant species, occurring in rocky shores and fouling communities from habitats with moderately low salinity to oceanic conditions [15–17]. In natural habitats, *R*. *clavigera* is an important predatory species structuring intertidal communities where it preys on a wide number of invertebrates, such as bivalves, limpets, chitons, barnacles and serpulid polychaetes [18–20]. *Reishia clavigera* preys upon bivalves by boring through the bivalve shell (chemo-mechanical process) [16, 17]. The high dietary plasticity of *R*. *clavigera*, which can change according to its ontogenetic stage and/or to spatial and temporal prey availability [17], suggests that it could have an important role in controlling the spread of invasive invertebrates.

In Hong Kong, the introduced bivalves *Xenostrobus securis* (Lamarck, 1819) and *Mytilopsis sallei* (Récluz, 1849) have become invasive only in some estuarine fouling communities [21, 22]. A previous study indicated that these invasive bivalves tolerate a greater range of salinities than the native counterpart *Brachidontes variabilis* (Krauss, 1848), which could explain their dominance under estuarine conditions [23]. The wide environmental tolerance of these invasive species suggests that they have the ability to spread in habitats with moderately low salinity and oceanic conditions. In the 1980’s *M*. *sallei* dominated fouling communities under oceanic conditions [24], but it is currently restricted to estuarine and disturbed environments [25]. The invasion of *X*. *securis* in estuaries in Europe could be favored by low predation compared to a native mussel [26]. In Hong Kong several studies have demonstrated that predatory gastropods play an important role on bivalve communities [17, 19, 27]; however, their potential role in predation on invasive bivalves remains undetermined.

The aim of this study was to compare the survival of the invasive bivalves *Xenostrobus securis* and *Mytilopsis sallei* and the native *Brachidontes variabilis* exposed to the native whelk *Reishia clavigera* under laboratory conditions with either normal or moderately low salinity as well as field conditions. Field experiments also tested the survival of the bivalves to potential predators located in two fouling communities, one pier located in a disturbed site (i.e., low salinity and poor water quality) with the absence of *R*. *clavigera* and another pier under oceanic conditions with *R*. *clavigera*. This study provides information about the potential role of the predatory whelk in limiting the spread of the invasive bivalves to other communities and its use as a biological control agent for the management of invasive bivalves.

## Materials and methods

### Species collection and laboratory conditions

**Fig 1:**
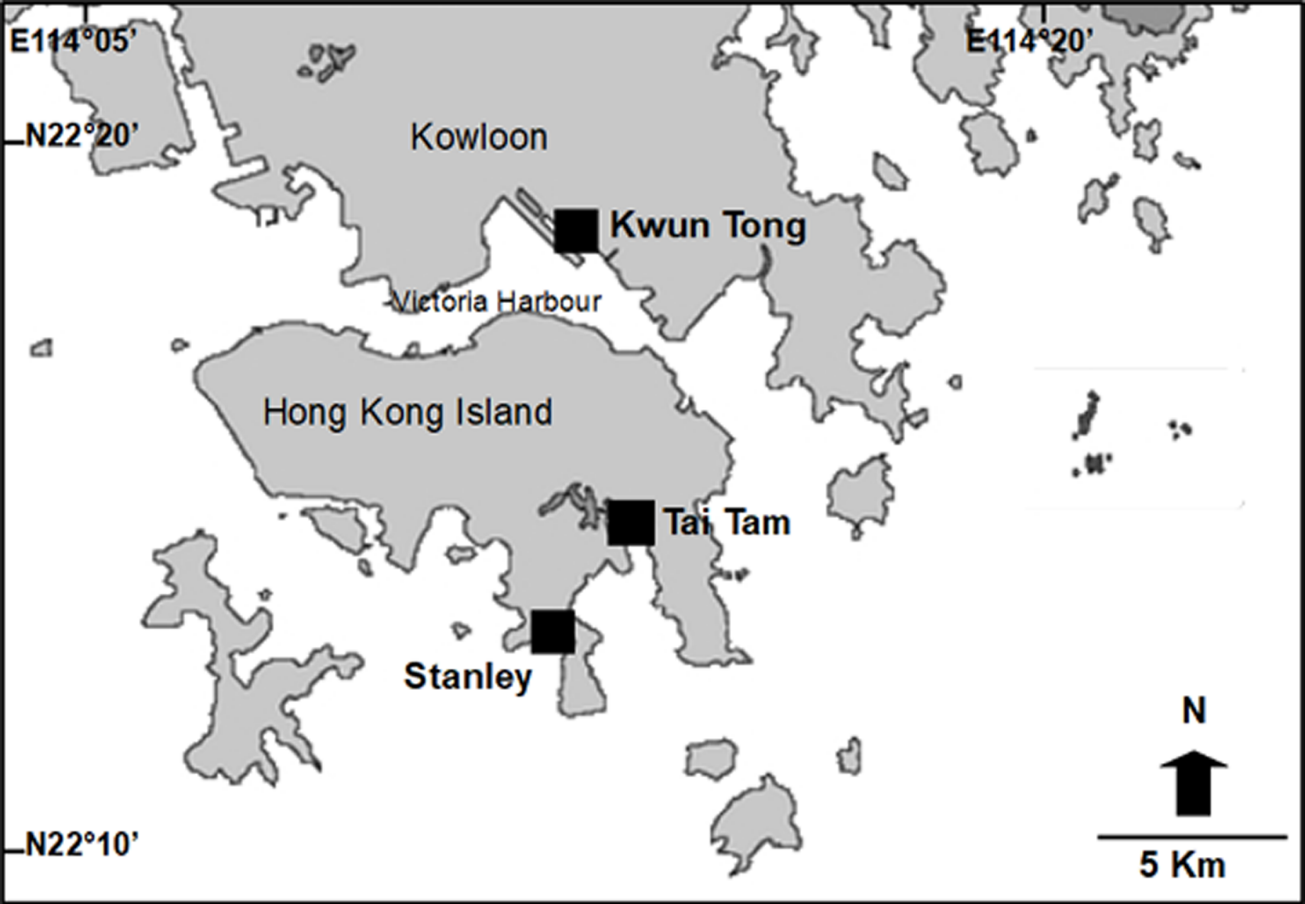
Map of the study sites. Filled squares indicate where the bivalves were collected and experiments conducted.

The native whelk *Reishia clavigera* was collected from the low intertidal and shallow subtidal in Stanley Pier. The invasive bivalves *Xenostrobus securis* and *Mytilopsis sallei* were collected from submerged substrates in the shallow subtidal zone under the pier located in Kwun Tong typhoon shelter. The native bivalve *B*. *variabilis* was collected from buoys within ~30 cm below the sea surface in Tai Tam bay (see Fig 1). No specific permissions were required for animal collection in this study because the species collected for the experiments are not endangered or protected species and were collected from public piers and buoys that are not protected. *Xenostrobus securis* and *B*. *variabilis*, both of which belong to the family Mytilidae, differ taxonomically at the subclass level with *M*. *sallei*, which belongs to the family Dreissenidae. In the laboratory, all species were sorted and kept separately in mesh cages in outdoor tanks (under natural photoperiod) of 100 L with constant seawater supply (sand filtered) and aeration. Temperatures and salinities of the running seawater during the experimental period ranged from 29 to 32°C and from 31 to 35‰, respectively. Experimental bivalve individuals ranged from 1.0 to 1.5 cm in shell length, whereas the native predatory whelk *R*. *clavigera* ranged from 2.5 to 3.5 cm in shell length. This size range of the bivalves represented the most common size in the field among the three species. The bivalves were fed with the microalgae *Chaetoceros gracilis* and with commercial coral food made of phytoplankton (Plancto, Aqua Medic, Germany). During the acclimation period, *R*. *clavigera* were fed with crushed individuals of the three bivalve species to ensure the predator recognized all prey species [26]. Each individual of the bivalve species and *R*. *clavigera* were used only for one experiment of this study to assure independency of the data.

Ten individuals of each bivalve species, within the range of 1.0 to 2.0 cm in shell length, were dried and used to measure the maximum length (L), height (H) and width (W), total individual biomass (TB), shell biomass (SB) and posterior adductor muscle biomass (AB). These measurements were used to determine the ratios of H to L, W to L, AB to TB, and shell thickness index (STI) to describe the morphology of the bivalve species. High H:L ratios (near 1) indicated round shaped shells, high W:L ratios indicated cupped shells, and high AB:TB ratios indicated that the bivalve had a larger adductor muscle in proportion to the total biomass. Higher values for TSI indicated thicker shells. TSI was calculated with the equation described in Fitzer et. al. (2015) [28].

### Laboratory experiments

Based on pilot observations, *Reishia clavigera* preyed on bivalves when there was more than one whelk per aquarium and the aquarium had developed biofilm on the walls. *Reishia clavigera* preyed on the bivalves by boring the edge of the shells. To ensure that *R*. *clavigera* fed on the bivalves, the aquaria (3.5 L) were under experimental conditions with running seawater for 5 days before starting the experiments for biofilm development. Two days before starting the experiments, three individuals of *R*. *clavigera* were introduced into the predation treatment aquaria for acclimation without food. All the aquaria were covered with plastic mesh (0.5 x 0.5 cm) to avoid the escape of the whelks. The survival of the invasive and native bivalves exposed to *R*. *clavigera* was tested under single choice and multiple choice experiments as recommended by Underwood and Clarke (2005) [29] to determine prey preference by the predator. The single choice experiments provided data for the prey consumption of each species, which was used to estimate the expected proportion of prey species consumed at random in the multiple choice experiment. Prey consumption may vary between species because predators may catch and handle prey species differently. Therefore, a preference by the predator would be demonstrated when the proportion of prey species consumed differed to the expectation derived from the single choice experiment.

Single choice experiment: Twelve individuals of a single bivalve species were attached to a PVC panel (7 x 15 cm) with plastic glue. Individuals were carefully glued only by the left valve to allow the bivalves to open their shells. All the panels were kept in tanks with running seawater for 24-h before the predation experiment to remove any glue residual. Each panel with a single bivalve species was introduced to either the predation treatment (with 3 randomly selected *Reishia clavigera*) or the control treatment aquaria (without predator to determine handling mortality). The experiment was under running seawater and each treatment had 6 replicates (∑*n* = 2 predation treatments × 3 bivalve species × 6 replicates = 36 experimental units). After 24-h, the number of surviving bivalves in each aquarium was recorded. The bivalves were considered alive when they reacted to the touch on the shell or the valves were tightly closed. Dead bivalves were easily determined because their valves were open with no flesh, whereas few killed bivalves (but not eaten) were open and did not react to touching.

Multiple choice experiments: Experimental conditions were similar to the single choice experiment; however, in this experiment 6 individuals of each bivalve species were randomly attached to one panel (i.e., 3 species × 6 individuals = 18 individuals per panel). Panels were then exposed to the predators (11 replicates; each with 3 *Reishia clavigera*) or to the control without predators (5 replicates) for 24-h to determine the survival number of the bivalves (i.e., 16 experimental units). The number of replicates for the control treatment in this experiment was lower than predation treatment because in the single experiment none of the bivalves in the control treatment died.

Acute temperature and salinity test on *Reishia clavigera*: The survival of *R*. *clavigera* was assessed through a 96-h acute temperature and salinity test to examine tolerance before performing the predation experiments under different salinity conditions. *Reishia clavigera* individuals were acclimated in indoor conditions for two weeks, in a 20 L plastic aquarium under 12:12 day/night photoperiod, filtered seawater (0.22 μm) with 30 ± 2‰ and 22 ± 1°C (indoor room temperature) and constant air supply (via aeration). Every second day whelks were fed with the bivalve *Xenostrobus securis* and the seawater was renewed. Feeding stopped 24-h before starting the test. Five whelks were placed in a 0.45 L aquarium. Survival was tested under a combination of three temperatures (14, 22 and 30°C) and three salinities (12, 22, 32‰), in an orthogonal and balanced design with 3 replicates (∑*n* = 3 temperatures × 3 salinities × 3 replicates = 27 experimental units). Each aquarium was covered with a transparent plastic film with small holes and provided with constant air supply using a Pasteur pipette. Seawater was renewed once at 48-h. Survival was monitored every 24-h until the end of the 96-h experiment. Whelks were considered dead when they were not attached and did not react to the touch of their foot muscle.

Multiple choice experiments in two salinity conditions: A second multiple choice experiment (similar setup to the previous multiple choice experiment) was performed under salinities of 22‰ and 32‰. Predation of the whelks on the bivalves was tested under these two salinities because superficial salinity in Kwun Tong typhoon shelter (with high abundance of invasive species) generally ranges from 19.5 to 32.6‰, and these two salinities are within the salinity tolerance range of the bivalves [23]. All the aquaria had running seawater for 5 days before the experiment for biofilm development. At 48-h before the experiment, the whelks were introduced to the tank and the salinity was adjusted to the experimental salinity in a stepwise manner (±2‰ per h). Aquaria had constant air supply and seawater was only renewed every 24-h to keep salinity stable. The panels were either exposed to predation by *Reishia clavigera* or to no predation (as control for experimental conditions without predators) under the two salinity treatments (22 and 32‰) with 6 replicates each (∑*n* = 2 predation treatments x 2 salinities x 6 replicates = 24 experimental units). The number of bivalve survivors was counted after 24-h.

### Field predation experiment

A field predation experiment was conducted in Kwun Tong and Stanley piers to determine predation under field conditions (Fig 1). These piers were chosen because of the contrasting conditions: fouling communities in Kwun Tong Pier, where *Reishia clavigera* was absent, was dominated by both invasive bivalve species, whereas in Stanley Pier, where *R*. *clavigera* was abundant, both invasive bivalve species were absent. Preliminarily 20 quadrats (25 × 25 cm) per pier (low intertidal) were sampled, finding that *R*. *clavigera* was absent in Kwun Tong Pier, whereas in Stanley pier its abundance was on average 50.4 (SD = 69.5) individuals per m^2^. In the present experiment, five individuals of each of the three bivalve species (total 15 individuals) were attached to a PVC panel (18 × 18 cm). Panels were randomly used for one of the three following treatments: 1) Predation by *R*. *clavigera* (bivalves exposed to three individuals of *R*. *clavigera*), 2) Open panel (bivalves exposed to any predator occurring in the site) and 3) control with predators excluded (∑*n* = 2 sites x 3 treatments x 9 replicates = 45 experimental units). Predation by *R*. *clavigera* and the control were achieved by enclosing the panels with plastic cages (kitchen sieves with mesh of 0.3 x 2.2 cm), whereas panels without cages were exposed to any predator occurring in the study site. Panels were randomly attached to the submerged vertical columns (i.e., pillars) of the piers in the low intertidal zone. After 5 days the panels were collected and the bivalve survival rates were examined and recorded. The temperature and salinity recorded in the field were 26.6°C and 11.2‰ at Kwun Tong and 26.9°C and 27.9‰ at Stanley on the first day, and 28.0°C and 24.5‰ at Kuwn Tong and 30.0°C and 23.4% at Stanley on the fifth day.

### Data analysis

To determine morphological differences among the bivalves species, the ratios of H:L, W:L, AB:TB and STI were compared with the one-way analysis of variance (ANOVA) tests. To determine whether *Reishia clavigera* prey randomly on the three bivalve species (no prey selection) the analysis was conducted as suggested by Underwood and Clarke (2005). The number of bivalves eaten in the single choice experiment (stage 1) and the number of bivalves eaten in the multiple choice experiment (stage 2) were used to derive the maximal likelihood estimators (for more details of the test, see Underwood and Clarke, 2005 [29]). Thirty-three random pairs of experimental units of predation on stage 1 (including three bivalve species) and predation on stage 2 were chosen for comparisons. The observed number of bivalves eaten was compared with the expected number using a *X*^*2*^ test with *k-1* degree of freedom (*k* = number of bivalve species).

A one-way ANOVA was used to compare the survival among bivalve species (fixed, 3 levels) for the non-choice and multiple choice experiments. Because none of the bivalves died under control conditions (without whelks), the control was not included in the analyses. A two-way ANOVA was used to compare the survival among bivalve species (fixed, 3 levels) and salinity (fixed, 2 levels). As only one individual of *Xenostrobus securis* died under 22‰, the control was not included in the data analyses.

For the field experiment, a two-way ANOVA was conducted to compare survival of the 3 bivalve species (fixed, 3 levels) exposed to *R*. *clavigera* between sites (fixed, 2 levels). The survival of the bivalves was also compared using a two-way ANOVA among the three bivalve species (fixed, 3 levels) and between the predation treatments (fixed, 2 levels, open vs. control panels) for each site separately. A Tukey’s HSD test was conducted for post-hoc comparison when factors were significantly different. Normality and homoscedasticity of variance were checked with Shapiro-Wilk tests and Levene tests, respectively. In cases when the data violated the homoscedasticity assumption for the ANOVA (in most cases due to 0 or 100% survival in some datasets) and no transformation was possible, we conducted the tests with raw data and the alpha value was decreased to 0.01 to reduce Type I error [30].

## Results

For morphological features of the bivalve species, the results of H:L ratios suggested that *Mytilopsis sallei* have the roundest shells (Table 1). The results of W:L ratios indicated that *M*. *sallei* and *Brachidontes variabilis* are more cupped than *Xenostrobus securis* (Table 1). The results of AB:TB ratios and STI indicated that *B*. *variabilis* have the largest adductor muscles (proportionally to total biomass) and the thickest shells, whereas *X*. *securis* have the smallest adductor muscles and thinnest shells among the bivalve species (Table 1).

**Table 1:** Summary of morphological features of the bivalve species *Xenostrobus securis*, *Mytilopsis sallei* and *Brachidontes variabilis* presented as average ratios of maximum shell height (H) and width (W) to length (L), adductor muscle biomass (AB) to total biomass (TB) and average of shell thickness index (STI). Values in brackets indicate the range of minimum and maximum values while a bolded *P* value indicate statistically significant difference among the three species in the corresponding parameter (by ANOVA tests).

	*X*. *securis*	*M*. *sallei*	*B*. *variabilis*	*P*
Size (cm)	16.74	13.84	14.81	
	(15.10–18.88)	(10.06–17.56)	(11.57–18.15)	
H:L	0.52	0.58	0.51	**<0.001**
	(0.47–0.55)	(0.52–0.65)	(0.48–0.54)	
W:L	0.38	0.43	0.44	**<0.001**
	(0.33–0.43)	(0.38–0.47)	(0.41–0.51)	
AB:TB	0.014	0.015	0.021	**0.032**
	(0.006–0.028)	(0.009–0.027)	(0.015–0.034)	
STI	0.59	0.65	0.72	**0.033**
	(0.48–0.76)	(0.50–0.89)	(0.53–0.91)	

Twenty out of the 33 *X*^*2*^ test comparisons, between observed and expected predation for stage 1 (single choice) and stage 2 (multiple choice), showed no significant differences (Table 2). However, the 13 remaining comparisons had significant differences. The overall results indicated that in most of the cases the whelk did not show a particular preference for any of the three bivalve species.

**Table 2:** Results of Chi-square tests to determine prey preference by *Reishia clavigera* on the bivalves *Xenostrobus securis* (*Xs*), *Mytilopsis sallei* (*Ms*) and *Brachidontes variabilis* (*Bv*).

Test	Obs	Obs	Obs	Obs	Obs	Obs	Exp	Exp	Exp	Exp	Exp	Exp	*X*^*2*^	*P*
	*Xs*	*Ms*	*Bv*	*Xs*	*Ms*	*Bv*	*Xs*	*Ms*	*Bv*	*Xs*	*Ms*	*Bv*		
	S1	S1	S1	S2	S2	S2	S1	S1	S1	S2	S2	S2		
1	12	12	3	6	0	0	12.0	12.0	2.5	2.7	2.7	0.6	7.35	>0.05
2	8	12	3	4	6	0	8.1	12.0	2.2	2.2	3.2	0.6	4.78	>0.10
3	12	12	4	6	5	4	12.0	12.0	4.9	2.5	2.5	1.0	16.42	**<0.005**
4	9	1	1	4	0	0	9.1	0.7	0.7	5.2	0.4	0.4	1.28	>0.10
5	12	11	1	5	4	0	12.0	11.0	0.7	3.0	2.8	0.2	2.09	>0.10
6	8	12	1	3	2	0	8.3	12.0	0.8	2.4	3.4	0.2	1.04	>0.10
7	8	12	3	6	5	1	8.5	12.0	2.7	2.2	3.1	0.7	7.94	**<0.05**
8	12	12	4	6	4	3	12.0	12.0	4.6	2.5	2.5	1.0	10.08	**<0.025**
9	12	11	1	6	4	0	12.0	10.9	0.7	3.0	2.8	0.2	3.71	>0.10
10	12	12	3	6	6	6	12.0	12.0	4.8	2.5	2.5	1.0	35.48	**<0.005**
11	9	1	1	6	5	0	8.4	2.8	0.5	4.3	1.4	0.3	11.45	**<0.01**
12	12	11	1	6	0	0	12.0	10.7	0.8	3.1	2.7	0.2	5.81	>0.10
13	12	12	3	4	6	0	12.0	12.0	2.3	2.7	2.7	0.5	5.21	>0.10
14	12	11	1	6	5	4	12.0	10.9	2.8	2.8	2.5	0.7	24.24	**<0.005**
15	8	12	3	4	0	0	8.7	12.0	2.6	2.2	3.1	0.7	5.26	>0.10
16	9	1	1	5	4	0	8.5	2.6	0.6	4.4	1.3	0.3	7.10	>0.05
17	12	12	4	3	2	0	12.0	12.0	3.5	2.6	2.6	0.8	1.03	>0.10
18	12	12	3	6	5	1	12.0	12.0	2.8	2.7	2.7	0.6	6.32	>0.05
19	8	12	3	6	4	3	8.4	12.0	3.7	2.1	3.0	0.9	12.47	**<0.01**
20	12	12	4	6	4	0	12.0	12.0	3.1	2.7	2.7	0.7	5.84	>0.10
21	9	1	1	6	6	6	7.2	2.7	2.7	3.5	1.3	1.3	39.47	**<0.005**
22	12	11	1	6	5	0	12.0	11.0	0.7	3.0	2.8	0.2	4.96	>0.10
23	9	12	3	6	0	0	9.7	12.0	2.5	2.4	3.0	0.6	9.13	**<0.05**
24	12	12	1	4	6	0	12.0	12.0	0.7	2.9	2.9	0.2	3.96	>0.10
25	12	1	1	6	5	4	12.0	2.9	2.5	4.1	1.0	0.9	30.51	**<0.005**
26	9	11	3	4	0	0	9.5	10.8	2.6	2.5	2.8	0.7	4.52	>0.10
27	8	12	4	5	4	0	8.5	12.0	3.1	2.2	3.0	0.8	5.10	>0.10
28	12	11	3	3	2	0	12.0	11.0	2.6	2.8	2.6	0.6	0.81	>0.10
29	8	12	1	6	5	1	8.4	12.0	1.3	2.3	3.3	0.4	7.92	**<0.05**
30	12	1	3	6	4	3	12.0	2.6	3.4	4.0	0.9	1.1	16.28	**<0.005**
31	12	12	3	6	4	0	12.0	12.0	2.3	2.7	2.7	0.5	5.21	>0.10
32	9	12	4	6	6	6	9.0	12.0	5.3	2.0	2.7	1.2	30.72	**<0.005**
33	12	11	1	6	5	0	12.0	11.0	0.7	3.0	2.8	0.2	4.96	>0.10

The test compared observed (Obs) and expected (Exp) number of bivalves eaten in single choice (stage 1 = S1) and multiple choice experiments (stage 2 = S2). Probabilities in bold indicate prey preference.

**Fig 2:**
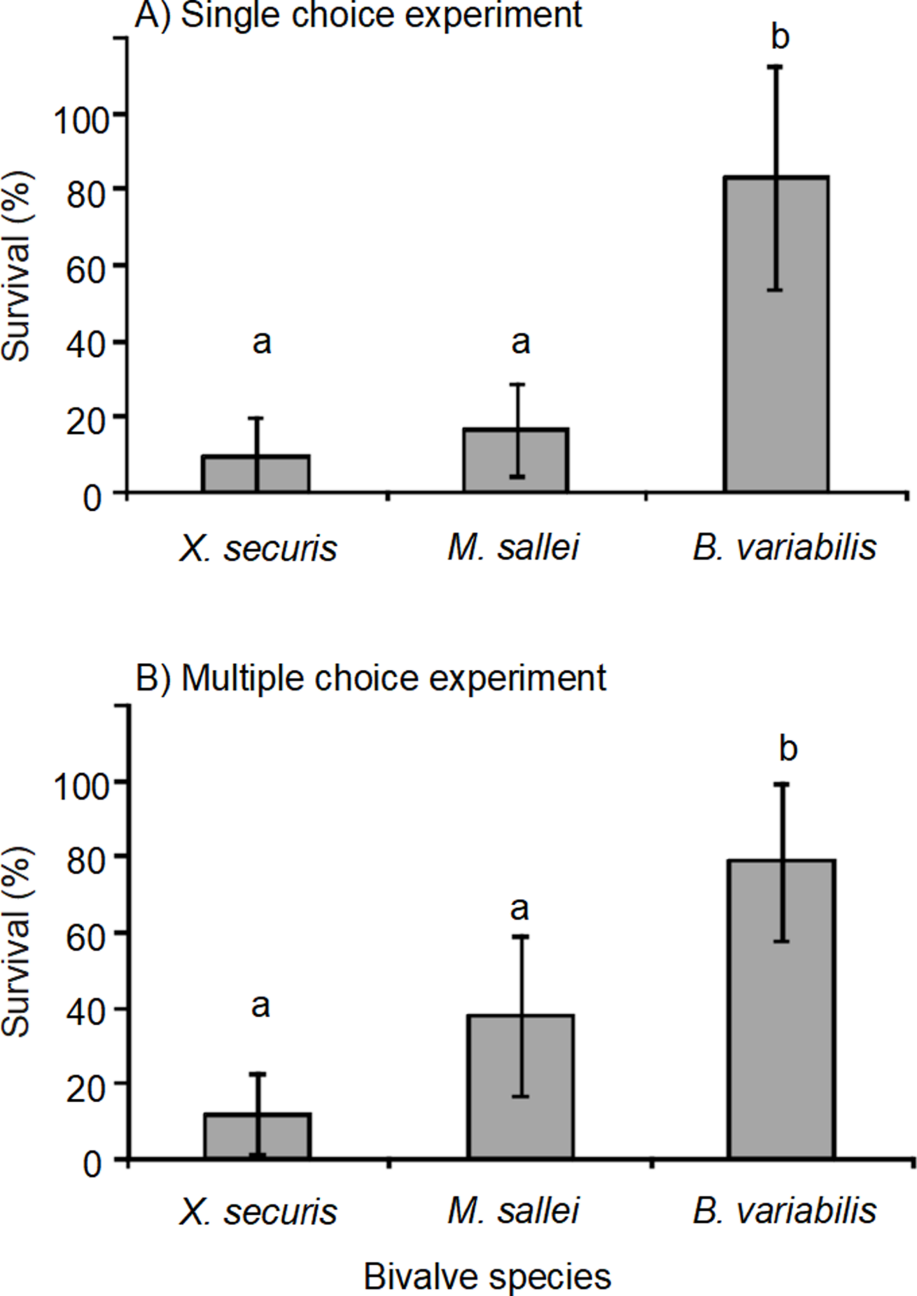
**Survival (± 95% CI) of the invasive bivalves *Xenostrobus securis* and *Mytilopsis sallei* alongside the native *Brachidontes variabilis* exposed to the predatory whelk *Reishia clavigera* in A) single choice experiment (stage 1) and B) multiple choice experiment (stage 2) for 24-h.** Bars with different lowercase letters indicate significantly different mean values (based on the ANOVA and the Tukey’s post-hoc tests; *P* < 0.05).

In the single choice experiment, survival of *Xenostrobus securis* and *Mytilopsis sallei* was significantly lower than that of *Brachidontes variabilis* after being exposed for 24-h to *Reishia clavigera* (Table 3A and Fig 2A). Less than 20% of the individuals of *X*. *securis* and *M*. *sallei* survived, whereas over 80% of *B*. *variabilis* survived. In the multiple choice experiments, the survival of the bivalves (exposed together with the whelks) showed a similar pattern as that observed in the single choice experiment. Survival of *X*. *securis* and *M*. *sallei* was significantly lower than *B*. *variabilis* (Table 3B and Fig 2B). *Xenostrobus securis* and *M*. *sallei* showed an average survival of 12 and 37% respectively, in contrast to 78% survival in *B*. *variabilis*.

**Table 3:** **Results of the ANOVA tests to compare the survival of the invasive bivalves *Xenostrobus securis* and *Mytilopsis sallei* and the native *Brachidontes variabilis* exposed to the predatory whelk *Reishia clavigera* in A) single choice experiment, B) multiple choice experiment and C) multiple choice experiment under two salinities for 24-h, and D) survival of *Reishia clavigera* after the 96-h acute temperature and salinity test**.

	*df*	MS	*F*	*P*
A) Single choice				
Species	2	142.72	16.88	**< 0.001**
Error	15	8.46		
B) Multiple choice				
Species	2	44.76	13.09	**< 0.001**
Error	30	3.42		
C) Multiple choice under two salinities				
Species	2	19.08	9.76	**0.001**
Salinity	1	21.78	11.14	**0.002**
Sp x Sa	2	8.69	4.45	**0.020**
Error	30	1.96		
				
D) Acute test on *R*. *clavigera*				
Temperature	2	2.70	3.48	0.053
Salinity	2	7.81	10.05	**0.001**
Temp x Sa	4	3.37	4.33	0.013
Error	18	0.78		

Acute test analysis was conducted with raw data and alpha value reduced to 0.01 due to heterogeneity of variances. Bold *P*-values indicate significant differences.

**Fig 3:**
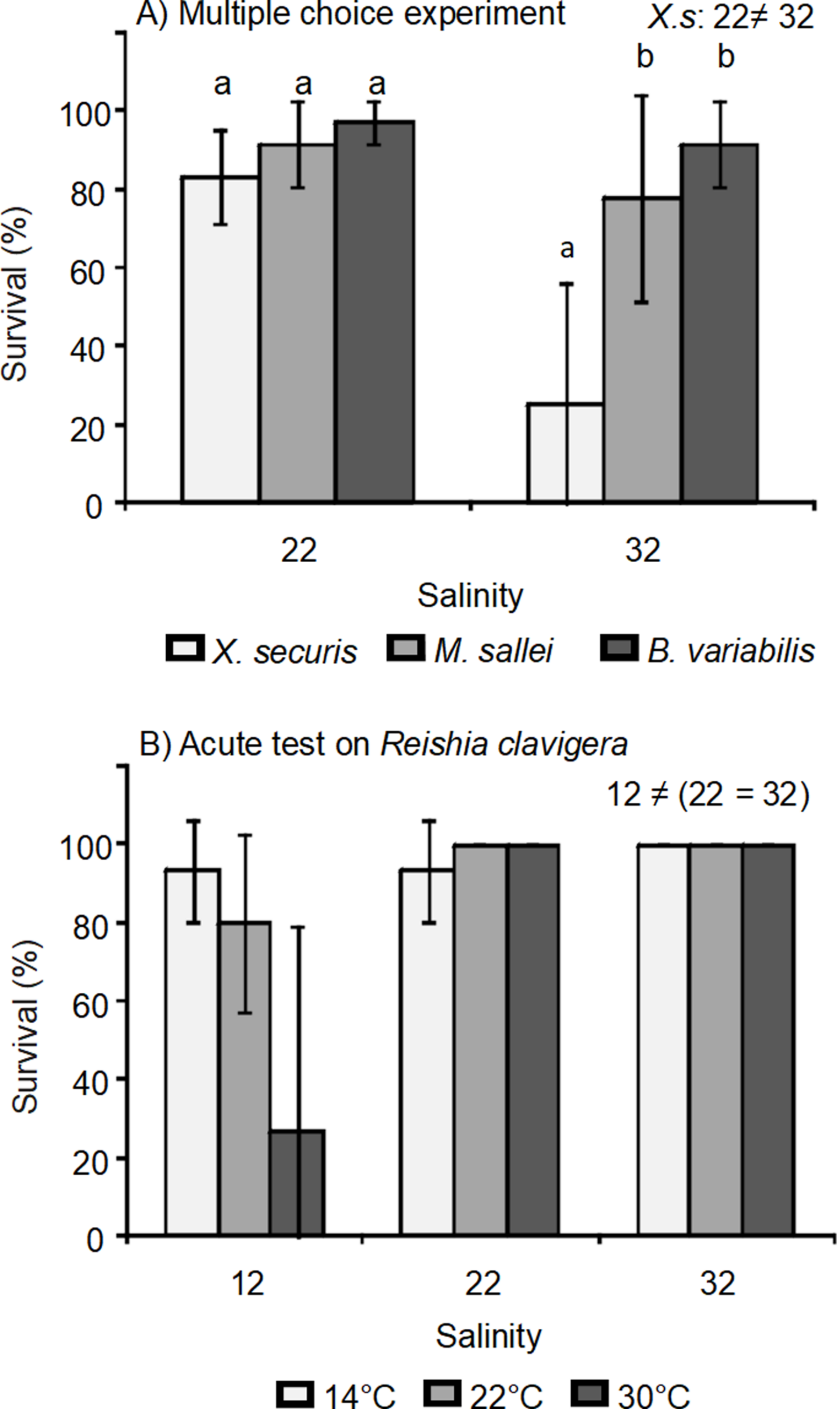
**A) Survival of the invasive bivalves to the predatory whelk *Reishia clavigera* under salinities of 22 and 32‰ for 24-h and B) survival of *Reishia clavigera* after the 96-h acute temperature and salinity test.** Error bars indicate ± 95% CI. Letters on the columns indicate the results of the Tukey’s post-hoc test for differences among species within each salinity treatment. Numbers on the right corner indicate differences between salinity treatments. *X*.*s = Xenostrobus securis*.

Multiple choice experiments under the two salinity conditions showed that the survival of the bivalve species was significantly affected by the interaction of salinity and bivalve species (Table 3C and Fig 3A). Post hoc analyses indicated that survival of *Xenostrobus securis* (25%) was significantly lower than *Mytilopsis sallei* (77%) and *Brachidontes variabilis* (91%) under salinity treatment of 32‰, but bivalve species survival did not significantly differ (survival above 83%) under salinity of 22‰ (Fig 3).

The results of the acute temperature and salinity test indicated that the survival of *Reishia clavigera* was significantly reduced by low salinity (12‰), in particular at 30°C (Table 3D and Fig 3B). At 22‰, the survival of the whelk was only reduced at 14°C while no mortality was observed at 32‰ across all temperature treatments.

**Fig 4:**
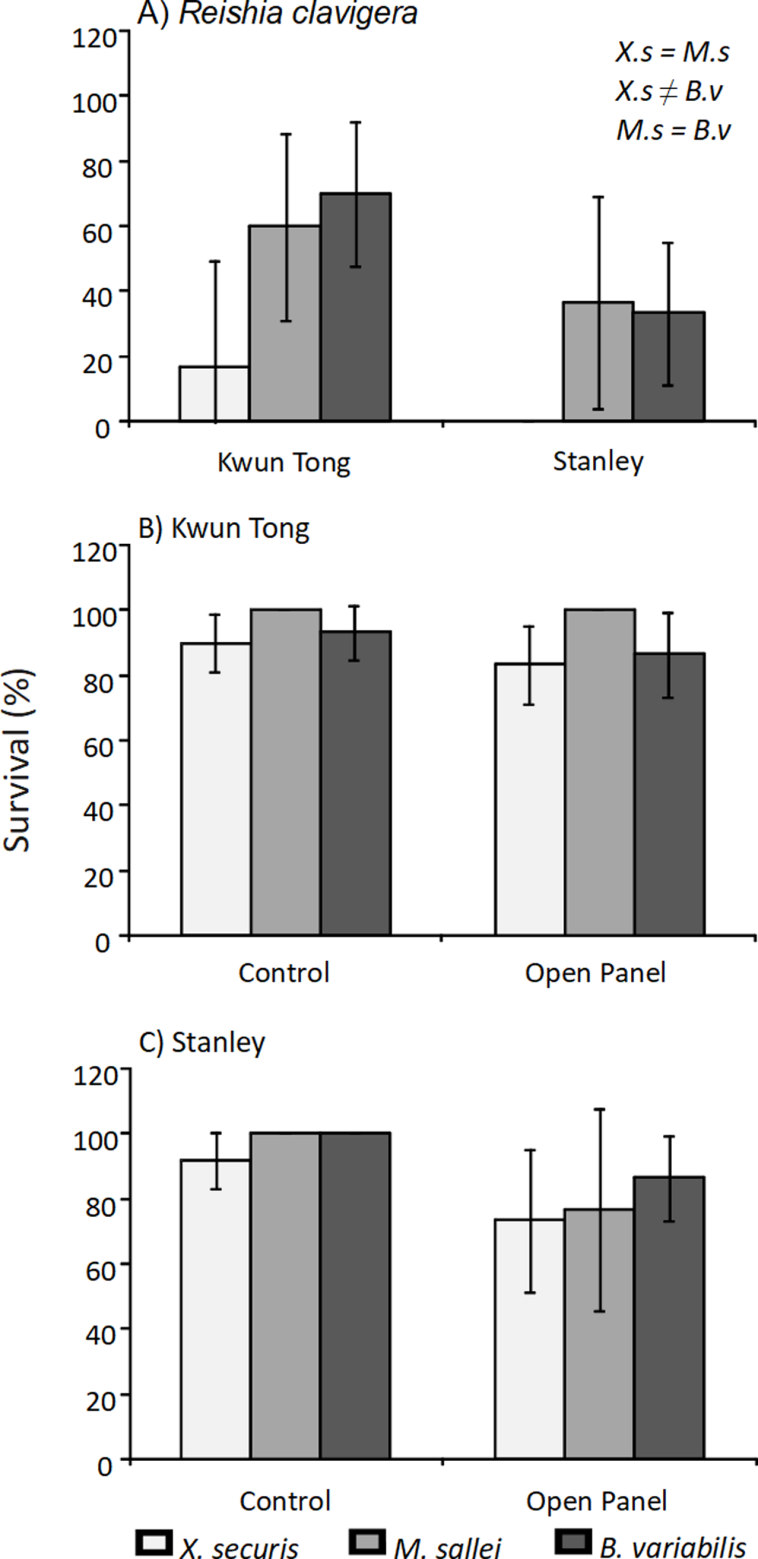
**Survival (± 95% CI) of bivalve species (*Xenostrobus securis*, *Mytilopsis sallei* and *Brachidontes variabilis*) exposed to A) *Reishia clavigera* at Kwun Tong and Stanley piers, and exposed to natural predators (i.e., open panels and control panels with predator excluded) in B) Kwun Tong pier and C) Stanley pier.** Letters on the right corner indicate the results of the Tukey’s post-hoc tests between species.

All whelks used in the field experiment survived throughout the experimental period. There were dead bivalves and few missing bivalves on the open panels. Possible missing bivalves were removed by predators because in the control panels all bivalves were still attached after the exposure period. Survival of the bivalves exposed to *Reishia clavigera* in the field was significantly different among the three bivalve species (Table 4A and Fig 4A). In general, *Xenostrobus securis* had the lowest survival among the bivalve species, with 16% survival at Kwun Tong and 0% at Stanley. *Mytilopsis sallei* had a survival of 60% at Kwun Tong and 36% at Stanley, whereas *Brachidontes variabilis* survival was 70% at Kwun Tong and 33% at Stanley. Though survival tended to be lower at Stanley Pier, there was no statistically significant difference in the results between the two sites (Table 4A and Fig 4A).

**Table 4:** Results of two-way ANOVA tests to compare the survival of the bivalve species (*Xenostrobus securis*, *Mytilopsis sallei* and *Brachidontes variabilis*) exposed to A) *Reishia clavigera* at Kwun Tong and Stanley piers, and exposed to natural predators (i.e., open panels and control panels with predator excluded) in B) Kwun Tong pier and C) Stanley pier.

	*df*	MS	*F*	*P*
A) *Reishia clavigera*				
Sites	1	14.69	5.76	0.023
Species	2	17.44	6.84	**0.004**
Si x Sp	2	0.78	0.31	0.739
Error	30	2.55		
B) Kwun Tong pier				
Species	2	1.44	4.81	0.015
Treatments	1	0.44	1.48	0.233
Sp x Tr	2	0.11	0.37	0.694
Error	30	0.30		
C) Stanley pier				
Species	2	0.75	0.69	0.512
Treatments	1	8.03	7.34	0.011
Sp x Tr	2	0.19	0.18	0.838
Error	30	1.09		

Tests were conducted with raw data and alpha value reduced to 0.01 due to heterogeneity of variances. The bold *P*-value indicates significant differences.

Survival of the bivalves on the open panels exposed to natural predators on fouling communities did not statistically differ from the control treatments in both Kwun Tong and Stanley pier (Table 4B and 4C and Fig 4B and 4C). However, *X*. *securis* generally had the lowest average survival in the open panel treatments among the three bivalve species, about 83% in Kwun Tong and 73% in Stanley pier, whereas the survival in control treatments was above 90% for all the species in both sites.

## Discussion

The results of this study clearly indicate that both invasive bivalves, *Xenostrobus securis* and *Mytilopsis sallei*, are more vulnerable than the native *Brachidontes variabilis* to predation by the whelk *Reishia clavigera*. Moreover, the similar survival of bivalves obtained from both single choice and multiple choice experiments indicate that the whelk probably does not have a preference for any particular bivalve species [29].

The low survival of the invasive species could be caused by a lack of effective defense and anti-predatory adaptation to the predatory whelk [18, 31, 32]. Anti-predatory responses in bivalves are diverse and these could vary from: changes in size and thickness of the shells [18], enlargement of the adductor muscle [33], increase of byssal thread production [34], reduction of clearance rate to lower chemical cues that attract predators [35], and movements and aggregation with other bivalves [36]. This predation experiment exposed the bivalves without previous exposure to the predator and hence did not establish anti-predatory responses. *Reishia clavigera* attacks bivalves by boring the shell via a chemo-mechanical process [16, 17]. Based on our observations, *R*. *clavigera* preyed on bivalves by boring the edge of the valves. The shell thickness index indicated that *Xenostrobus securis* had the thinner shell followed by *Mytilopsis sallei* and *Brachidontes variabilis*. Consequently, *R*. *clavigera* may spend less time handling the invasive bivalves, which could explain their low survival in the experiments. In contrast, *B*. *variabilis* has a thicker shell, as well as a crenulated valve margin (absent in the invasive bivalves) that could provide a better defense against the whelks. In this case, invasive bivalves may require to thicken their shells to avoid predation by *R*. *clavigera*.

The survival of *Reishia clavigera* under the acute temperature and salinity test is consistent with its distribution in Hong Kong [16]. As showed in the current study, the lowest survival of *R*. *clavigera* was under the combination of 12‰ and 30°C. Populations could, therefore, be drastically affected by prolonged drops in salinity during summer (rainy season). In the present survival experiment under two salinity conditions, predation on bivalve species decreased in moderately low salinity treatments, where *R*. *clavigera* likely became stressed under salinity of 22‰. However, sub-lethal responses should be measured to corroborate environmental stress on the whelk in further studies.

Studies have found that physical habitats and environmental conditions modify predator-prey interactions between benthic species [37, 38]. Recruitment and survival of the invasive bivalve *Musculista senhousia* in a California estuary, for example, is higher under low salinity where predators cannot access them [4]. Therefore, the interaction between *Reishia clavigera* and the bivalves must be affected by moderate low salinities in the field, which could explain the current distribution of the invasive bivalves in estuarine environments in Hong Kong [21].

As demonstrated in this study, *Xenostrobus securis* would have benefited the most by low salinity conditions, increasing its survival from 25% (at 32‰) to 83% (at 22‰). Low salinities in summer season could reduce predation pressure on communities inhabited by *Reishia clavigera*. *Xenostrobus securis* larvae, which develop normally in salinities of 8–17‰ [39], may spread to surrounding areas during low salinity events, but their long-term establishment will depend on predation and competition pressure in those communities. Interestingly, this study showed that *Mytilopsis sallei* had a higher average survival under 32‰ in the salinity experiment compared to the survival in the previous single and multiple choice experiments (Figs 1 and 2). This difference could be caused by the static seawater used in the salinity experiment. A previous acute temperature and salinity test on these bivalves indicated that *X*. *securis* has higher clearance rate than *M*. *sallei* [23]. High clearance rate is related to a higher release of chemical cues that attract predators [35]. Under static seawater conditions, *X*. *securis* may attract *R*. *clavigera*, releasing predation pressure on *M*. *sallei*, which could indicate some level of prey preference.

The predation pattern of *Reishia clavigera* on bivalves is very similar between the laboratory and field experiments as revealed in this study. Consistently, *Xenostrobus securis* had the lowest average survival compared to *Mytilopsis sallei* and *Brachidontes variabilis*. *Xenostrobus securis* seemed to have better survival at the Kwun Tong typhoon shelter than at Stanley pier (Fig 4A). The lower seawater quality (lower salinity, hypoxia, sedimentation, pollution, etc) in Kwun Tong typhoon shelter [40] likely reduced the predation efficacy of *R*. *clavigera*.

In the present field study, the survival of bivalves in open panels exposed to natural predators did not statistically differ from the controls after 5 days of exposure. In contrast, a similar field experiment carried out in an intertidal rocky shore found that the survival of exotic bivalves decreased to about 30% in 3 days [41]. The high abundance of fouling species (i.e., high prey availability) on the piers in Hong Kong [42] may have reduced the opportunity or need of predators to attack the experimental bivalves. Predation on fouling communities can also be lower than on natural reefs [32]. *Reishia clavigera* consumes *Brachidontes variabilis*, but it also preys on a wide range of other species showing high diet plasticity [17]. Hence, food availability and preference under natural conditions must be investigated to further understand the role of *R*. *clavigera* on invasive species.

## Conclusion

Increasing attention has been given to the use of native predators as biological control agents for fouling communities and invasive species [10, 12]. A successful agent species must have life history traits suitable for the habitat and target invasive species as its prey [43]. This study demonstrated that the whelk *Reishia clavigera* preyed on the invasive *Xenostrobus securis* and *Mytilopsis sallei* more often than on the native *Brachidontes variabilis*. However, this predation pattern could be due to the predation vulnerability of the invasive bivalves rather than a prey preference exercised by *R*. *clavigera*. Although *R*. *clavigera* is a common species on rocky shores and in fouling communities on piers, predation on invasive bivalves is reduced when salinity decreases. Hence, our conclusion is that *R*. *clavigera* may not be the most suitable bio-control agent for invasive bivalves in Hong Kong marine communities. Nevertheless, bivalves have a wide range of predators, such as whelks, sea stars, crabs, fishes and birds [34, 36, 41] that limit their abundance and distribution. Hong Kong, as a hotspot of biodiversity [14], has several species of predatory gastropods and crabs that could prey upon invasive bivalves [16] in concurrence with *R*. *clavigera* to control their abundance and distribution.
